# Statistical analysis on finger replacement schemes for RAKE receivers in the soft handover region with multiple BSs over i.n.d. fading channels

**DOI:** 10.1371/journal.pone.0179126

**Published:** 2017-06-12

**Authors:** Sung Sik Nam, Young-Chai Ko, Mohamed-Slim Alouini, Seyeong Choi

**Affiliations:** 1 School of Electrical Engineering, Korea University, Seoul, Korea; 2 Department of Electrical Engineering, KAUST, Thuwal, Makkah Province, Saudi Arabia; 3 Department of Information and Communication Engineering, Wonkwang University, Jeonbuk, Korea; Tongji University, CHINA

## Abstract

A new finger replacement technique which is applicable for RAKE receivers in the soft handover region has been proposed and studied under the ideal assumption that the fading is both independent and identically distributed from path to path. To supplement our previous work, we present a general comprehensive framework for the performance assessment of the proposed finger replacement schemes operating over independent but non-identically distributed (i.n.d.) faded paths. To accomplish this object, we derive new closed-form expressions for the target key statistics which are composed of i.n.d. exponential random variables. With these new expressions, the performance analysis of various wireless communication systems over more practical channel environments can be possible.

## Introduction

Multipath fading is an unavoidable physical phenomenon that affects considerably the performance of wideband wireless communication systems. While usually viewed as a deteriorating factor, multipath fading can also be exploited to improve the performance by using RAKE-type receivers. RAKE receiver is designed to optimally detect a signal transmitted over a dispersive multi-path channel. It is an extension of the concept of the matched filter. In the RAKE receiver, one RAKE finger is assigned to each multi-path, thus maximizing the amount of received signal energy. Each of these different paths are combined to form a composite signal that is expected to have substantially better characteristics for the purpose of demodulation than just a single path. However, in the soft handover (SHO) region, due to the limited number of fingers in the mobile unit, we are faced with a problem of how to judiciously select a subset of paths for RAKE reception to achieve the required performance.

Finger replacement techniques for RAKE reception in the SHO region have been proposed and analyzed over independent and identically distributed (i.i.d.) fading environments with two base stations (BSs) in [1] which was extended to the case of multiple BSs in [2]. The proposed schemes in [2] are basically based on the block comparison among groups of resolvable paths from different BSs and lead to the reduction of complexity while offering commensurate performance in comparison with previously proposed schemes in [3, 4]. However, in practice, the i.i.d. fading scenario on the diversity paths is not always realistic due to, for example, the different adjacent multipath routes with the same path loss and the resulting unbalance among paths. This non-identical scenario may induce non-negligible performance degradation compared with the results for i.i.d. fading scenario and it may eventually lead to affect in determining system parameters (e.g., the number of combined paths). Although this non-identical consideration is important from a practical standpoint, [2] was able to investigate the effect of the non-uniform power delay profile of the finger replacement schemes only with computer simulations due to the high complexity of the analysis. The major difficulties lie in deriving the target statistics with non-identical parameters. Although the applied conditional probability density function (PDF) based approach used in [5] for deriving the required key statistics is quite impressive and contributive, the analytical framework in [5] was limited to i.i.d. Rayleigh fading assumptions.

With this observation in mind, in this work we mathematically attack these main difficulties. More specifically, we address the key mathematical formalisms which are the statistics of partial sums and the two-dimensional joint statistics of partial sums of the i.n.d. ordered random variables (RVs). Then, the mathematical analysis framework given in [2] is slightly modified suitable for these newly derived joint statistical results. Note that our derived results are much simpler than the original results with the multiple-fold integral forms based on the conventional moment generating function (MGF) based approaches. Note also that it is configured to be directly applicable to other applications and other fading scenarios.

It is very noticeable that the finger replacement schemes in [1, 2] can also apply to the “trendy” applications such as millimeter-wave (mmWave) communication systems [6–8] in which an antenna diversity scheme operates like a finger replacement scheme. In mmWave systems, with an increase of the number of Rake fingers, a significant improvement is expected because the channel impulse response is completely decayed in a very short time period compared with the typical RAKE receiver based systems (i.e., carrier frequencies below 10 GHz). Therefore, a larger number of fingers are required while there exist the limited number of fingers in the mobile unit. This can point to the very clear conclusion that it is more necessary to apply the low complexity and low power consumption finger management schemes with a minimal amount of additional network resources for RAKE reception in the SHO region with multiple base stations to achieve the required performance. In addition, for mmWave communication systems where antenna arrays are employed, the antenna elements may receive line-of-sight signals with different Rayleigh fading factor because these signals may take completely different (independent) propagation paths before arriving at the receiver [9]. Therefore, the non-identical condition assumed in this work is worth considering in terms of contribution.

Note that it is very important to accurately characterize the performance of wireless communication systems over more practical channel models as for examples in [10–12]. From this perspective, we believe that even though the overall analytical framework is based on the previous result in [2], the new results contained in this paper are quite suitable, general and contributive for the practical channel models in that the approach used in [2] even based on the i.i.d. fading scenario requires some case-specific manipulations.

The rest of this paper is organized as follows. In the ‘System Models and Performance Measures’ section, we present the system models as well as the mode of operation of the finger replacement scheme under consideration and provide the results of a general comprehensive framework for the outage performance based on the statistical results over i.n.d. fading channels. We then provide in the ‘Key Statistics’ section, some closed-form expressions of the required key statistics. Final section provides some simulation results and concluding remarks.

## System models and performance measures

Among the path scanning schemes proposed in [2], we consider the full scanning method. With this method, if the combined output signal-to-noise ratio (SNR) of current assigned fingers is greater than a certain target SNR, a one-way SHO is used and no finger replacement is needed. Otherwise, the receiver attempts a two-way SHO by starting to scan additional paths from the serving BS as well as all the target BSs.

**Fig 1:**
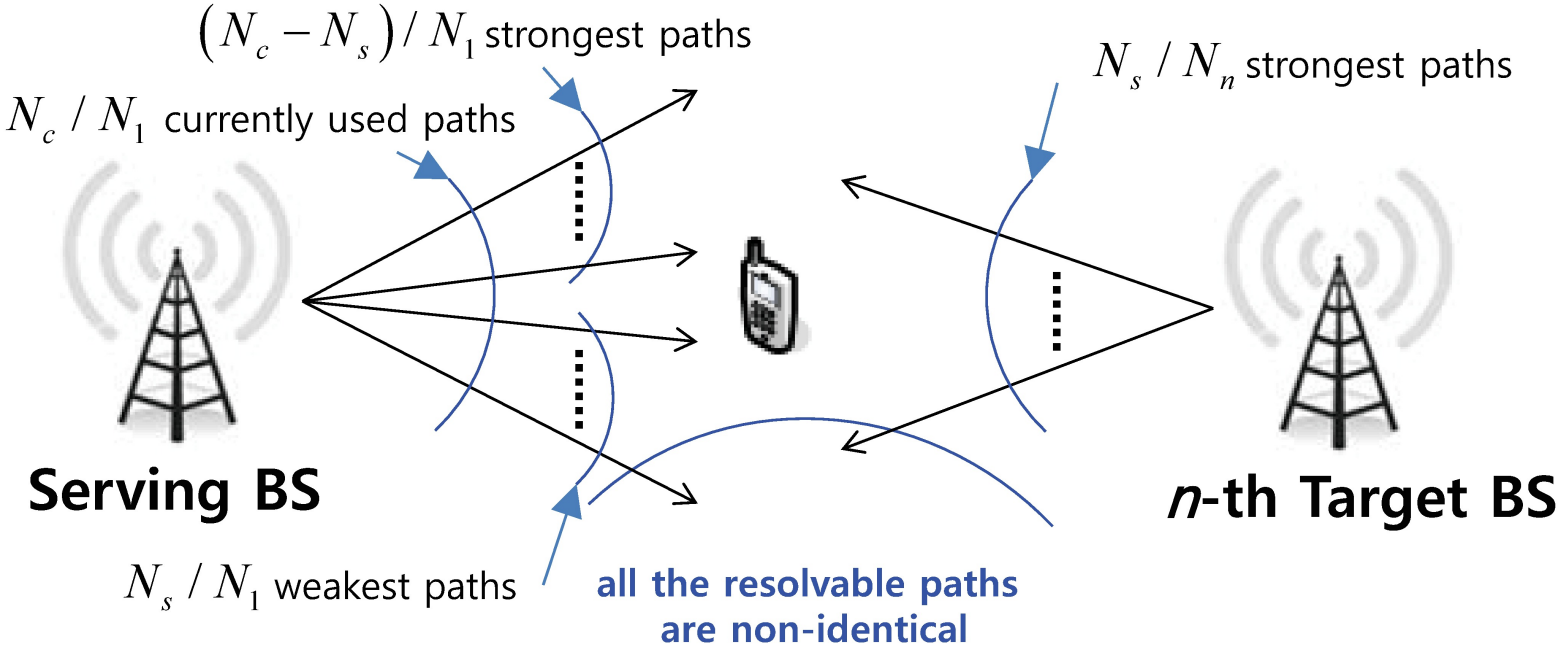
Finger replacement schemes for RAKE receivers in the soft handover region.

We assume that *L* BSs are active, and there are a total of *N*_(*L*)_ resolvable paths where $N_{\left(L\right)}=\sum_{n=1}^{L}N_{n}$ and *N*_*n*_ is the number of resolvable paths from the *n*-th BS. In the SHO region as shown in Fig 1, only *N*_*c*_ out of *N*_(*n*)_ (1 ≤ *n* ≤ *L*) paths are used for RAKE reception. Without loss of generality, let *N*_1_ be the number of resolvable paths from the serving BS and *N*_2_, *N*_3_, ⋯, *N*_*L*_ be those from the target BSs. In the SHO region, the receiver is assumed at first to rely only on *N*_1_ resolvable paths and, as such, starts with *N*_*c*_/*N*_1_-generalized selection combining (GSC) [13] which combines the strongest *N*_*c*_ resolvable paths among the *N*_1_ available ones. These schemes are based on the comparison of blocks consisting of *N*_*s*_(< *N*_*c*_ < *N*_*n*_) paths from each BS.

Let *u*_*i*, *n*_ (*i* = 1, 2, ⋯, *N*_*n*_) be the *i*-th order statistics out of *N*_*n*_ SNRs of paths from the *n*-th BS by arranging *N*_*n*_ non-negative i.n.d. RVs, ${\left\{\gamma_{j,n}\right\}}_{j=1}^{N_{n}}$, where *γ*_*j*,*n*_ is the SNR of the *j*-th path from the *n*-th BS, in decreasing order of magnitude such that *u*_1,*n*_ ≥ *u*_2,*n*_ ≥ ⋯ ≥ *u*_*N*_*n*_,*n*_. If we let *Y* be the sum of the *N*_*c*_ − *N*_*s*_ strongest paths from the serving BS and *W*_*n*_ be the sum of the *N*_*s*_ smallest paths from the serving BS for *n* = 1 and be the sum of the *N*_*s*_ strongest paths from the target BS for *n* = 2, ⋯, *L* as

$$\begin{array}{c} Y=\sum_{i=1}^{N_{c}-N_{s}}u_{i,1} \end{array}$$ (1)

and

$$\begin{array}{c} W_{n}=\left\{\begin{array}{c} \sum_{i=N_{c}-N_{s}+1}^{N_{c}}u_{i,n},\;\;\;\;n=1;\\ \sum_{i=1}^{N_{s}}u_{i,n},\;\;\;\;\;\;\;\;n=2,\cdots ,L, \end{array}\right. \end{array}$$ (2)

then, the received output SNR after GSC is given by *Y* + *W*_1_. At the beginning of every time slot, the receiver compares the GSC output SNR, *Y* + *W*_1_, with a certain target SNR. If *Y* + *W*_1_ is greater than or equal to the target SNR, a one-way SHO is used and no finger replacement is needed. On the other hand, whenever *Y* + *W*_1_ falls below the target SNR, the receiver attempts a two-way SHO by starting to scan additional paths from the target BSs.

To study the performance of the finger replacement scheme for i.n.d. fading assumptions, we look into the outage performance. Based on the mode of operation in [2], an overall outage probability is declared when the final combined SNR, *γ*_*F*_, falls below a predetermined threshold, *x*, as

$$\begin{array}{c} F_{\gamma_{F}}\left(x\right)=\text{Pr}\left[\gamma_{F}<x\right] \end{array}$$ (3)

where

$$\begin{array}{c} \gamma_{F}=\left\{\begin{array}{cc} Y+W_{1}, & Y+W_{1}\geq \gamma_{T};\\ Y+\text{max}\left\{W_{1},W_{2},\cdots ,W_{L}\right\}, & Y+W_{1}<\gamma_{T}. \end{array}\right. \end{array}$$ (4)

Considering two cases that i) the final combined SNR is greater than or equal to the target SNR, *γ*_*T*_, (i.e., *x* ≥ *γ*_*T*_) and ii) the final combined SNR falls below the target SNR, (i.e., 0 < *x* < *γ*_*T*_), separately, we can rewrite Eq (3) as

$$\begin{array}{c} F_{\gamma_{F}}\left(x\right)=\left\{\begin{array}{cc} \text{Pr}\left[Y+\text{max}\left\{W_{1},W_{2},\cdots ,W_{L}\right\}<x\right], & 0<x<\gamma_{T};\\ \text{Pr}\left[\gamma_{T}\leq Y+W_{1}<x\right]\\ +\text{Pr}\left[Y+W_{1}<\gamma_{T},\gamma_{T}\leq Y+\text{max}\left\{W_{1},W_{2},\cdots ,W_{L}\right\}<x\right], & x\geq \gamma_{T}. \end{array}\right. \end{array}$$ (5)

Note that the major difficulty in the analysis is to derive the required key statistics of ordered RVs. In [1] and [2], with an i.i.d. assumption, the required statistics were obtained by applying the conditional PDF based approach proposed in [5]. Since our concerns are more practical channel models in which the average SNRs of each path (or branch) are different, unlike the i.i.d. case we need to consider realistic frequency selective channels which have a non-uniform delay profile (i.e., exponentially decaying power delay profile(PDP)). However, the proposed method in [5] can not be directly adopted in the case of i.n.d. fading environments here.

Recently, the unified framework to determine the joint statistics of partial sums of ordered i.i.d. RVs has been introduced in [14] which shows that the required key statistics of any partial sums of ordered RVs can be systematically obtained in terms of the MGF and the PDF. In [15, 16], some extended works from the mathematical approach proposed in [14] to i.n.d. fading channels can be found. In this paper, with the help of [14–16], the required key statistics to investigate the outage probability in Eq (5) over i.n.d. fading channels will be obtained.

Note that based on the mode of operation, *Y* and *W*_1_ are correlated while *W*_*n*_ (for *n* = 2, ⋯, *L*) is independent of *Y*. Hence, three probabilities in Eq (5) can be expressed as

$$\begin{array}{l} \text{Pr}\left[Y+\text{max}\left\{W_{1},W_{2},\cdots ,W_{L}\right\}<x\right]\\ =\int_{0}^{x}\int_{0}^{x-y}\cdots \int_{0}^{x-y}f_{Y,W_{1},W_{2},\cdots ,W_{L}}\left(y,w_{1},w_{2},\cdots ,w_{L}\right)dw_{L}\cdots dw_{2}dw_{1}dy\\ =\int_{0}^{x}\int_{0}^{x-y}\cdots \int_{0}^{x-y}f_{Y,W_{1}}\left(y,w_{1}\right)f_{W_{2}}\left(w_{2}\right)\cdots f_{W_{L}}\left(w_{L}\right)dw_{L}\cdots dw_{2}dw_{1}dy\\ =\int_{0}^{x}\int_{0}^{x-y}f_{Y,W_{1}}\left(y,w_{1}\right)\int_{0}^{x-y}f_{W_{2}}\left(w_{2}\right)dw_{2}\cdots \int_{0}^{x-y}f_{W_{L}}\left(w_{L}\right)dw_{L}\cdots dw_{1}dy\\ =\int_{0}^{x}\int_{0}^{x-y}f_{Y,W_{1}}\left(y,w_{1}\right)\prod_{n=2}^{L}F_{W_{n}}\left(x-y\right)dw_{1}dy, \end{array}$$ (6)

$$\begin{array}{ccc} & & \text{Pr}\left[\gamma_{T}\leq Y+W_{1}<x\right]=F_{Y+W_{1}}\left(x\right)-F_{Y+W_{1}}\left(\gamma_{T}\right), \end{array}$$ (7)

and

$$\begin{array}{l} \text{Pr}\left[Y+W_{1}<\gamma_{T},\gamma_{T}\leq Y+\text{max}\left\{W_{1},W_{2},\cdots ,W_{L}\right\}<x\right]\\ =\int_{0}^{\gamma_{T}}\int_{0}^{\gamma_{T}-y}f_{Y,W_{1}}\left(y,w_{1}\right)\int_{0}^{x-y}f_{W_{2}}\left(w_{2}\right)dw_{2}\cdots \int_{0}^{x-y}f_{W_{L}}\left(w_{L}\right)dw_{L}\cdots dw_{1}dy\\ =\int_{0}^{\gamma_{T}}\int_{0}^{\gamma_{T}-y}f_{Y,W_{1}}\left(y,w_{1}\right)\prod_{n=2}^{L}F_{W_{n}}\left(x-y\right)dw_{1}dy. \end{array}$$ (8)

Similar to the identical case in [2], it is also very important to study the complexity of finger replacement schemes over i.n.d. case by accurately quantifying the performance measures such as the average number of path estimations, the average number of SNR comparisons, and the SHO overhead, which are required during the SHO process of these schemes over i.n.d. case. Note that with these performance measures, a comprehensive investigation of the tradeoff between complexity and performance over i.n.d. fading channels can be feasible. These important design parameters can be evaluated by directly applying the defined formulas presented in [2] with the required key statistics for i.n.d. ordered RVs which will be derived in this work. Hence, based on the mathematical approach proposed in [14–16], we here focus on the derivation of the following key statistics such as the cumulative distribution function (CDF) [17] of the *N*_*c*_/*N*_1_-GSC output SNR, $F_{Y+W_{1}}(\cdot )$, the 2-dimensional joint PDF of two adjacent partial sums, *Y* and *W*_1_, of order statistics, $f_{Y+W_{1}}(\cdot ,\cdot )$, and the CDF of the sum of the *N*_*s*_ strongest paths from each target BS, $F_{W_{n}}(\cdot )$ (2 ≤ *n* ≤ *L*).

## Key statistics

In this section, we introduce the key statistics which are essential to solve Eqs (6), (7), and (8). More specifically, in these three cases, only the best *N*_*c*_ or *N*_*s*_ among *N*_*n*_ (*N*_*s*_ ≤ *N*_*c*_ ≤ *N*_*n*_) ordered RVs are involved in the partial sums. Thus, based on the unified frame work in [14] and the extended work for i.n.d. case in [15, 16], each key statistics for three cases can be derived by applying the special step approach based on the substituted groups instead of original groups for each cases (i.e., starting from 2-dimensional joint statistics, 4-dimensional joint statistics, and 2-dimensional joint statistics, respectively) as follows;

1. $F_{Y+W_{1}}(x)$: In this case, *Y* and *W*_1_ have the following relationship
  $$\begin{array}{c} \overset{Y}{\overset{︷}{u_{1,1},\cdots ,u_{N_{c}-N_{s}-1,1},u_{N_{c}-N_{s},1}}},\overset{W_{1}}{\overset{︷}{u_{N_{c}-N_{s}+1,1},\cdots ,u_{N_{c}-1,1},u_{N_{c},1}}},u_{N_{c}+1,1},\cdots ,u_{N,1}. \end{array}$$ (9)
  Here, by adopting the unified-frame work in [14], the target CDF of interest, $F_{Y+W_{1}}(x)$, can be obtained from the transformed higher dimensional joint PDFs where *Z*_1_ and *Z*_2_ have the following relationships
  $$\begin{array}{c} \underset{Z_{1}}{\underset{︸}{u_{1,1},\cdots ,u_{N_{c}-N_{s}-1,1},u_{N_{c}-N_{s},1},u_{N_{c}-N_{s}+1,1},\cdots ,u_{N_{c}-1,1}}},\underset{Z_{2}}{\underset{︸}{u_{N_{c},1}}},u_{N_{c}+1,1},\cdots ,u_{N,1}. \end{array}$$ (10)
  Therefore, if we let *Z*′ = *Y*+*W*_1_ where $Y+W_{1}=\sum_{i=1}^{N_{c}}u_{i,1}$ for convenience, then $Z_{2}<\frac{Z^{\prime }}{N_{c}}$ and as a result, we can derive the target CDF of *Z*′ with the 2-dimensional joint PDF of $Z_{1}=\sum_{i=1}^{N_{c}-1}u_{i,1}$ and $Z_{2}=u_{N_{c}}{}_{,1}$ as
  $$\begin{array}{ll} F_{Y+W_{1}}\left(x\right) & =\int_{0}^{x}f_{Z^{\prime }}\left(z\right)dz\\ & =\int_{0}^{x}\int_{0}^{\frac{z}{N_{c}}}f_{Z_{1},Z_{2}}\left(z-z_{2},z_{2}\right)dz_{2}dz. \end{array}$$ (11)
2. $f_{Y+W_{1}}(x,y)$: In this case, we can derive the target 2-dimensional PDF of $Y=\sum_{i=1}^{N_{c}-N_{s}}u_{i,1}$ and $W_{1}=\sum_{i=N_{c}-N_{s}+1}^{N_{c}}u_{i,1}$ by transferring the 4-dimensional joint PDF of $Z_{1}=\sum_{i=1}^{N_{c}-N_{s}-1}u_{i,1}$, $Z_{2}=u_{N_{c}}{{}_{-}}_{N_{s},1}$, $Z_{3}=\sum_{i=N_{c}-N_{s}+1}^{N_{c}-1}u_{i,1}$, and $Z_{4}=u_{N_{c}}{}_{,1}$ where *Z*_1_, *Z*_2_, *Z*_3_, and *Z*_4_ have the following relationships
  $$\overset{Y}{\overset{︷}{\underset{Z_{1}}{\underset{︸}{u_{1,1},\cdots ,u_{N_{c}-N_{s}-1,1\;}}},\underset{Z_{2}}{\underset{︸}{u_{N_{c}-N_{s},1}}},}}\overset{W_{1}}{\overset{︷}{\underset{Z_{3}}{\underset{︸}{u_{N_{c}-N_{s}+1,1},\cdots ,u_{N_{c}-1,1}}},\underset{Z_{4}}{\underset{︸}{u_{N_{c},1}}}}},u_{N_{c}+1,1},\cdots ,u_{N,1}.$$ (12)
  From Eq (12), the following valid conditions can be directly obtained
  $$\begin{array}{c} Z_{1}\geq \left(N_{c}-N_{s}-1\right)Z_{2}\;\Rightarrow \;Z_{1}+Z_{2}\geq \left(N_{c}-N_{s}\right)Z_{2}, \end{array}$$ (13)
  $$\begin{array}{c} Z_{3}\geq \left(N_{s}-1\right)Z_{4}\;\Rightarrow \;Z_{3}+Z_{4}\geq N_{s}\cdot Z_{4}, \end{array}$$ (14)
  and
  $$\begin{array}{c} N_{s}\cdot Z_{2}\geq Z_{3}+Z_{4}. \end{array}$$ (15)
  Therefore, with the 4-dimensional joint PDF of *Z*_1_, *Z*_2_, *Z*_3_, and *Z*_4_, letting *X* = *Z*_1_ + *Z*_2_ and *Y* = *Z*_3_ + *Z*_4_, we can obtain the target 2-dimensional joint PDF of *Y* and *W*_1_ with the help of a function of a marginal PDF by integrating over *z*_2_ and *z*_4_ yielding
  $$\begin{array}{c} f_{Y,W_{1}}\left(x,y\right)=\int_{0}^{\frac{y}{N_{s}}}\int_{\frac{y}{N_{s}}}^{\frac{x}{N_{c}-N_{s}}}f_{Z_{1},Z_{2},Z_{3},Z_{4}}\left(x-z_{2},z_{2},y-z_{4},z_{4}\right)dz_{2}dz_{4}. \end{array}$$ (16)
3. $F_{W_{n}}(x)$ (2 ≤ *n* ≤ *L*): Similar to case 1), the target one-dimensional CDF of $W_{n}=\sum_{i=1}^{N_{s}}u_{i,n}$ with the 2-dimensional joint PDF of $Z_{1}^{\prime }=\sum_{i=1}^{N_{s}-1}u_{i,n}$ and $Z_{2}^{\prime }=u_{N_{s},n}$ can be derived with the help of a function of a marginal PDF as
  $$\begin{array}{c} F_{W_{n}}\left(x\right)=\int_{0}^{x}\int_{0}^{\frac{z}{{}_{N_{s}}}}f_{Z_{1}^{\prime },Z_{2}^{\prime }}\left(z-z_{2}^{\prime },z_{2}^{\prime }\right)dz_{2}^{\prime }dz. \end{array}$$ (17)

Note that the above novel generic results in Eqs (11), (16) and (17) are quite general and can be applied for any RVs. In here, we assume the i.n.d. RVs with a common exponential PDF and CDF given by

$$\begin{array}{c} p_{i_{l,n}}\left(x\right)=\frac{1}{{\bar{\gamma }}_{i_{l,n}}}\text{exp}\left(-\frac{x}{{\bar{\gamma }}_{i_{l,n}}}\right),\;x\geq 0 \end{array}$$ (18)

and

$$\begin{array}{c} P_{i_{l,n}}\left(x\right)=1-\text{exp}\left(-\frac{x}{{\bar{\gamma }}_{i_{l,n}}}\right),\;x\geq 0 \end{array}$$ (19)

respectively, where ${\bar{\gamma }}_{i_{l,n}}$ is the average of the *l*-th RV at *n*-th BS. In what follows, for convenience in a mathematical representation, we define the set of (*N*_*n*_ − *m* − 1)-tuples, *τ*_*n*_(*i*, *m*, *N*_*n*_), as $\tau_{n}(i,m,N_{n})=\{i_{m}{}_{,n},i_{m+1},n,\cdots ,i_{N_{n}}{}_{,n}\}$. We also assume, without loss of generality, that *N*_*n*_ = *N*, *u*_*i*,*n*_ = *u*_*i*_, and ${\bar{\gamma }}_{i_{l,n}}={\bar{\gamma }}_{i_{l}}$ for all *n* = 1, 2, ⋯, *L*. For the analytical tractability, we start from $F_{Y,W_{1}}(x,y)$.

### Joint PDF of two adjacent partial sums *Y* and *W*_1_ over i.n.d. Rayleigh fading, $f_{Y,W_{1}}(x,y)$

As shown in Eq (16), the target 2-dimensional joint PDF of interest, $f_{Y,W_{1}}(x,y)$, can be obtained from the transformed higher dimensional joint PDF. Based on the result in [16, Eq (54)], to evaluate the 2-fold integrations in Eq (16), the multiple product expression, $\prod_{j=N_{s}+1}^{N}(1-\text{exp}(-\frac{z_{4}}{{\bar{\gamma }}_{i_{j}}}))$, needs to be converted to the summation expression with the help of the property of exponential multiplication. Note that the product of two exponential numbers of the same base can be simply represented as the sum of the exponents with the same base. Let *N*_*s*_ + 1 = *n*_1_, *N* = *n*_2_, and $\text{exp}(-\frac{z_{4}}{{\bar{\gamma }}_{i_{j}}})=\text{exp}(-a_{i_{j}})$ for convenience, then

$$\begin{array}{c} \prod_{j=N_{s}+1}^{N}\left(1-\text{exp}\left(-\frac{z_{4}}{{\bar{\gamma }}_{i_{j}}}\right)\right)=\prod_{j=n_{1}}^{n_{2}}\left(1-\text{exp}\left(-a_{i_{j}}\right)\right). \end{array}$$ (20)

Starting from the case of *n*_1_ = 1 and *n*_2_ = 2, we extend this result to the general case for arbitrary *n*_1_ and *n*_2_. At first, let *n*_1_ = 1 and *n*_2_ = 2, then we can write Eq (20) as the following summation expression

$$\begin{array}{l} \prod_{j=1}^{2}\left(1-\text{exp}\left(-a_{i_{j}}\right)\right)=\left(1-\text{exp}\left(-a_{i_{1}}\right)\right)\left(1-\text{exp}\left(-a_{i_{2}}\right)\right)\\ =1-\text{exp}\left(-a_{i_{1}}\right)-\text{exp}\left(-a_{i_{2}}\right)+\text{exp}\left(-a_{i_{1}}-a_{i_{2}}\right). \end{array}$$ (21)

Similarly, for *n*_1_ = 1 and *n*_2_ = 3, we get

$$\begin{array}{l} \prod_{j=1}^{3}\left(1-\text{exp}\left(-a_{i_{j}}\right)\right)=\left(1-\text{exp}\left(-a_{i_{1}}\right)\right)\left(1-\text{exp}\left(-a_{i_{2}}\right)\right)\left(1-\text{exp}\left(-a_{i_{3}}\right)\right)\\ =1-\text{exp}\left(-a_{i_{1}}\right)-\text{exp}\left(-a_{i_{2}}\right)-\text{exp}\left(-a_{i_{3}}\right)+\text{exp}\left(-a_{i_{1}}-a_{i_{2}}\right)+\text{exp}\left(-a_{i_{1}}-a_{i_{3}}\right)\\ \;+\text{exp}\left(-a_{i_{2}}-a_{i_{3}}\right)-\text{exp}\left(-a_{i_{1}}-a_{i_{2}}-a_{i_{3}}\right). \end{array}$$ (22)

After simplification with a few manipulations, we can re-write the multiple product expressions in Eqs (21) and (22) as the following simplified summation expressions, respectively,

$$\begin{array}{ccc} & \prod_{j=1}^{2}\left(1-\text{exp}\left(-a_{i_{j}}\right)\right) & =1+\left(-1\right)\sum_{{j^{\prime }}_{1}=1}^{2}\text{exp}\left(-a_{i_{{j^{\prime }}_{1}}}\right)+{\left(-1\right)}^{2}\sum_{{j^{\prime }}_{1}=1}^{1}\sum_{{j^{\prime }}_{2}={j^{\prime }}_{1}+1}^{2}\text{exp}\left(-a_{i_{{j^{\prime }}_{1}}}-a_{i_{{j^{\prime }}_{2}}}\right), \end{array}$$ (23)

and

$$\begin{array}{l} \prod_{j=1}^{3}\left(1-\text{exp}\left(-a_{i_{j}}\right)\right)\\ =1+\left(-1\right)\sum_{{j^{\prime }}_{1}=1}^{3}\text{exp}\left(-a_{i_{{j^{\prime }}_{1}}}\right)+{\left(-1\right)}^{2}\sum_{{j^{\prime }}_{1}=1}^{2}\sum_{{j^{\prime }}_{2}={j^{\prime }}_{1}+1}^{3}\text{exp}\left(-a_{i_{{j^{\prime }}_{1}}}-a_{i_{{j^{\prime }}_{2}}}\right)\\ \;+{\left(-1\right)}^{3}\sum_{{j^{\prime }}_{1}=1}^{1}\sum_{{j^{\prime }}_{2}={j^{\prime }}_{1}+1}^{2}\sum_{{j^{\prime }}_{3}={j^{\prime }}_{2}+1}^{3}\text{exp}\left(-a_{i_{{j^{\prime }}_{1}}}-a_{i_{{j^{\prime }}_{2}}}-a_{i_{{j^{\prime }}_{3}}}\right). \end{array}$$ (24)

Finally, after simplifying and generalizing the above equations, we can obtain the generalized expression of Eq (20) as

$$\begin{array}{ccc} & \prod_{j=N_{s}+1}^{N}\left(1-\text{exp}\left(-\frac{z_{4}}{{\bar{\gamma }}_{i_{j}}}\right)\right) & =1+\sum_{g=1}^{N-N_{s}}{\left(-1\right)}^{g}\sum_{{j^{\prime }}_{1}={j^{\prime }}_{0}+N_{s}+1}^{N-g+1}\cdots \sum_{{j^{\prime }}_{g}={j^{\prime }}_{g-1}+1}^{N}\text{exp}\left(-\sum_{m=1}^{g}\frac{z_{4}}{{\bar{\gamma }}_{i_{{j^{\prime }}_{m}}}}\right). \end{array}$$ (25)

Note that with the help of Eq (25), the 4-dimensional joint PDF given in [16, Eq (45)] can be expressed in the summation form which makes Eq (16) more tractable. Hence, inserting the rewritten expression of [16, Eq (45)] into Eq (16), after some manipulations, we can rewrite Eq (16) as

$$\begin{array}{l} f_{Y,W_{1}}\;\left(\;x,y\;\right)=\;\sum_{\begin{array}{c} i_{N_{c}},\cdots ,i_{N_{1}}\\ i_{N_{c}}\neq \cdots \neq i_{N_{1}} \end{array}}^{1,2,\cdots ,N_{1}}\;\frac{1}{{\bar{\gamma }}_{i_{N_{c}}}}\;\sum_{\begin{array}{c} i_{N_{c}-N_{s}}=1\\ i_{N_{c}-N_{s}}\neq i_{N_{c},\cdots ,}i_{N_{1}} \end{array}}^{N_{1}}\;\frac{1}{{\bar{\gamma }}_{i_{N_{c}-N_{s}}}}\;\sum_{\begin{array}{c} \left\{\;i_{N_{c}-N_{s}+1},\cdots ,i_{N_{c}-1}\;\right\}\in \\ {\text{P}}_{N_{s}\;-\;1}\left(\;I_{N_{1}}\;-\;\left\{\;i_{N_{c}-N_{s}}\;\right\}\;-\;\left\{\;i_{N_{c}},\cdots ,i_{N_{1}}\;\right\}\;\right) \end{array}}\;\sum_{k=N_{c}-N_{s}+1}^{N_{c}-1}\;C_{k,N_{c}-N_{s}+1,N_{c}-1}\\ {}[\sum_{\begin{array}{c} \left\{\;i_{1},\cdots ,i_{N_{c}-N_{s}-1}\;\right\}\in \\ {\text{P}}_{N_{c}\;-\;N_{s}\;-\;1}\;\left(\;\begin{array}{c} I_{N_{1}}-\left\{\;i_{N_{c}-N_{s}}\;\right\}-\left\{\;i_{N_{c}},\cdots ,i_{N_{1}}\;\right\}\\ -\left\{\;i_{N_{c}-N_{s}+1},\cdots ,i_{N_{c}-1}\;\right\} \end{array}\;\right) \end{array}}\;\sum_{h=1}^{N_{c}-N_{s}-1}\;C_{h,1,N_{c}-N_{s}-1}\text{ exp }\left(\;-\;\frac{x}{{\bar{\gamma }}_{i_{h}}}\;\right)\text{ exp }\left(\;-\;\frac{y}{{\bar{\gamma }}_{i_{k}}}\;\right)\\ \times \;\int_{0}^{\frac{y}{N_{s}}}\;\int_{\frac{y}{N_{s}}}^{\frac{x}{N_{c}-N_{s}}}\;\text{exp }\left(\;-\;\left(\;\sum_{l=1}^{N_{c}-N_{s}}\;\left(\;\frac{1}{{\bar{\gamma }}_{i_{l}}}\;\right)\;-\;\frac{N_{c}-N_{s}}{{\bar{\gamma }}_{i_{h}}}\;\right)z_{2}\;\right)\text{ exp }\left(\;-\;\left(\;\sum_{l=N_{c}-N_{s}+1}^{N_{c}}\;\left(\;\frac{1}{{\bar{\gamma }}_{i_{l}}}\;\right)\;-\;\frac{\left(\;N_{s}\;\right)}{{\bar{\gamma }}_{i_{k}}}\;\right)z_{4}\;\right)U\;\left(\;x\;-\;\left(N_{c}\;-\;N_{s}\right)\cdot z_{2}\;\right)\;U\;\left(\;y\;-\;\left(\;N_{s}\;\right)\cdot z_{4}\;\right)dz_{2}dz_{4}\\ +\;\sum_{l=1}^{N_{s}-1}\;{\left(-1\right)}^{l}\;\sum_{j_{1}=j_{0}+N_{c}-N_{s}+1}^{N_{c}-l}\;\cdots \sum_{j_{l}=j_{l-1}+1}^{N_{c}-1}\;\;\sum_{\begin{array}{c} \left\{\;i_{1},\cdots ,i_{N_{c}-N_{s}-1}\;\right\}\in \\ {\text{P}}_{N_{c}\;-\;N_{s}\;-\;1}\;\left(\;\begin{array}{c} I_{N_{1}}\;-\;\left\{\;i_{N_{c}-N_{s}}\;\right\}\;-\;\left\{\;i_{N_{c}},\cdots ,i_{N_{1}}\;\right\}\;\\ -\;\left\{\;i_{N_{c}-N_{s}+1},\cdots ,i_{N_{c}-1}\;\right\} \end{array}\;\right) \end{array}}\sum_{h=1}^{N_{c}-N_{s}-1}C_{h,1,N_{c}-N_{s}-1}\text{ exp }\left(\;-\;\frac{x}{{\bar{\gamma }}_{i_{h}}}\;\right)\text{ exp }\left(\;-\;\frac{y}{{\bar{\gamma }}_{i_{k}}}\;\right)\\ \times \;\int_{0}^{\frac{y}{N_{s}}}\;\int_{\frac{y}{N_{s}}}^{\frac{x}{N_{c}-N_{s}}}\;\text{exp }\left(\;-\;\left(\;\sum_{l=1}^{N_{c}-N_{s}}\;\left(\;\frac{1}{{\bar{\gamma }}_{i_{l}}}\;\right)\;+\;\sum_{q=1}^{l}\;\frac{1}{{\bar{\gamma }}_{i_{j_{q}}}}\;-\;\frac{N_{c}-N_{s}}{{\bar{\gamma }}_{i_{h}}}\;-\;\frac{l}{{\bar{\gamma }}_{i_{k}}}\;\right)\;z_{2}\;\right)\text{ exp }\left(\;-\;\left(\;\sum_{l=N_{c}-N_{s}+1}^{N_{c}}\;\left(\;\frac{1}{{\bar{\gamma }}_{i_{l}}}\;\right)\;-\;\frac{\left(\;N_{s}-l\;\right)}{{\bar{\gamma }}_{i_{k}}}\;-\;\sum_{q=1}^{l}\;\frac{1}{{\bar{\gamma }}_{i_{j_{q}}}}\;\right)\;z_{4}\;\right)\\ U\left(x-\left(N_{c}\;-\;N_{s}\right)\cdot z_{2}\right)U\left(y-\left(l\cdot z_{2}+\left(N_{s}-l\right)\cdot z_{4}\right)\right)dz_{2}dz_{4}]\\ +\sum_{\begin{array}{c} i_{N_{c}},\cdots ,i_{N_{1}}\\ i_{N_{c}}\neq \cdots \neq i_{N_{1}} \end{array}}^{1,2,\cdots ,N_{1}}\frac{1}{{\bar{\gamma }}_{i_{N_{c}}}}\sum_{g=1}^{N_{1}-N_{c}}{\left(-1\right)}^{g}\sum_{j\prime_{1}=j\prime_{0}+N_{c}+1}^{N_{1}-g+1}\cdots \sum_{{j^{\prime }}_{g}={j^{\prime }}_{g-1}+1}^{N_{1}}\sum_{\begin{array}{c} i_{N_{c}-N_{s}}=1\\ i_{N_{c}-N_{s}}\neq i_{N_{c},\cdots ,}i_{N_{1}} \end{array}}^{N_{1}}\frac{1}{{\bar{\gamma }}_{i_{N_{c}-N_{s}}}}\\ \sum_{\begin{array}{c} \left\{i_{N_{c}-N_{s}+1},\cdots ,i_{N_{c}-1}\right\}\in \\ {\text{P}}_{N_{s}\;-\;1}\;\left(I_{N_{1}}-\left\{i_{N_{c}-N_{s}}\right\}-\left\{i_{N_{c}},\cdots ,i_{N_{1}}\right\}\right) \end{array}}\sum_{k=N_{c}-N_{s}+1}^{N_{c}-1}C_{k,N_{c}-N_{s}+1,N_{c}-1}\\ {}[\sum_{\begin{array}{c} \left\{\;i_{1},\cdots ,i_{N_{c}-N_{s}-1}\;\right\}\in \\ {\text{P}}_{N_{c}\;-\;N_{s}\;-\;1}\;\left(\;\begin{array}{c} I_{N_{1}}-\left\{\;i_{N_{c}-N_{s}}\;\right\}-\left\{\;i_{N_{c}},\cdots ,i_{N_{1}}\;\right\}\\ -\left\{\;i_{N_{c}-N_{s}+1},\cdots ,i_{N_{c}-1}\;\right\} \end{array}\;\right) \end{array}}\;\sum_{h=1}^{N_{c}-N_{s}-1}\;C_{h,1,N_{c}-N_{s}-1}\text{ exp }\left(\;-\;\frac{x}{{\bar{\gamma }}_{i_{h}}}\;\right)\text{ exp }\left(\;-\;\frac{y}{{\bar{\gamma }}_{i_{k}}}\;\right)\\ \times \int_{0}^{\frac{y}{N_{s}}}\int_{\frac{y}{N_{s}}}^{\frac{x}{N_{c}-N_{s}}}\text{exp}\left(-\left(\sum_{l=1}^{N_{c}-N_{s}}\left(\frac{1}{{\bar{\gamma }}_{i_{l}}}\right)-\frac{N_{c}-N_{s}}{{\bar{\gamma }}_{i_{h}}}\right)z_{2}\right)\text{exp}\left(-\left(\sum_{l=N_{c}-N_{s}+1}^{N_{c}}\left(\frac{1}{{\bar{\gamma }}_{i_{l}}}\right)+\sum_{m=1}^{g}\frac{1}{{\bar{\gamma }}_{i_{{j^{\prime }}_{m}}}}-\frac{\left(N_{s}\right)}{{\bar{\gamma }}_{i_{k}}}\right)z_{4}\right)\\ U\left(x-\left(N_{c}\;-\;N_{s}\right)\cdot z_{2}\right)U\left(y-\left(N_{s}\right)\cdot z_{4}\right)dz_{2}dz_{4}\\ +\;\sum_{l=1}^{N_{s}-1}\;{\left(-1\right)}^{l}\;\sum_{j_{1}=j_{0}+N_{c}-N_{s}+1}^{N_{c}-l}\;\cdots \;\sum_{j_{l}=j_{l-1}+\;1}^{N_{c}-1}\;\sum_{\begin{array}{c} \left\{\;i_{1},\cdots ,i_{N_{c}-N_{s}-1}\;\right\}\in \\ {\text{P}}_{N_{c}\;-\;N_{s}\;-\;1}\;\left(\;\begin{array}{c} I_{N_{1}}\;-\;\left\{\;i_{N_{c}-N_{s}}\;\right\}\;-\;\left\{\;i_{N_{c}},\cdots ,i_{N_{1}}\;\right\}\;\\ -\;\left\{\;i_{N_{c}-N_{s}+1},\cdots ,i_{N_{c}-1}\;\right\} \end{array}\;\right) \end{array}}\sum_{h=1}^{N_{c}-N_{s}-1}C_{h,1,N_{c}-N_{s}-1}\text{exp}\left(-\frac{x}{{\bar{\gamma }}_{i_{h}}}\right)\text{exp}\left(-\frac{y}{{\bar{\gamma }}_{i_{k}}}\right)\\ \times \;\int_{0}^{\frac{y}{N_{s}}}\;\int_{\frac{y}{N_{s}}}^{\frac{x}{N_{c}-N_{s}}}\;\text{exp }\left(\;-\;\left(\;\sum_{l=1}^{N_{c}-N_{s}}\;\left(\;\frac{1}{{\bar{\gamma }}_{i_{l}}}\;\right)\;+\;\sum_{q=1}^{l}\;\frac{1}{{\bar{\gamma }}_{i_{j_{q}}}}\;-\;\frac{N_{c}-N_{s}}{{\bar{\gamma }}_{i_{h}}}\;-\;\frac{l}{{\bar{\gamma }}_{i_{k}}}\;\right)\;z_{2}\;\right)\text{ exp }\left(\;-\;\left(\;\sum_{l=N_{c}-N_{s}+1}^{N_{c}}\;\left(\;\frac{1}{{\bar{\gamma }}_{i_{l}}}\;\right)\;+\;\sum_{m=1}^{g}\;\frac{1}{{\bar{\gamma }}_{i_{{j^{\prime }}_{m}}}}\;-\;\frac{\left(\;N_{s}-l\;\right)}{{\bar{\gamma }}_{i_{k}}}\;-\;\sum_{q=1}^{l}\;\frac{1}{{\bar{\gamma }}_{i_{j_{q}}}}\;\right)\;z_{4}\;\right)\\ U\left(x-\left(N_{c}\;-\;N_{s}\right)\cdot z_{2}\right)U\left(y-\left(l\cdot z_{2}+\left(N_{s}-l\right)\cdot z_{4}\right)\right)dz_{2}dz_{4}]. \end{array}$$ (26)

In Eq (26), the closed-form expressions for the first and the third integral terms can be obtained by simply applying the basic exponential integration [18] and using the following useful common function

$$\begin{array}{ll} \text{I}\left(x,e,a,b;y,f,c,d\right) & =\int_{c}^{d}\int_{a}^{b}\text{exp}\left(e\cdot x\right)\text{exp}\left(f\cdot y\right)dxdy\\ & =\frac{1}{e\cdot f}\left\{\text{exp}\left(e\cdot b\right)-\text{exp}\left(e\cdot a\right)\right\}\left\{\text{exp}\left(f\cdot d\right)-\text{exp}\left(f\cdot c\right)\right\}. \end{array}$$ (27)

With Eq (27), letting $\alpha =-\left(\sum_{l=N_{c}-N_{s}+1}^{N_{c}}\left(\frac{1}{{\bar{\gamma }}_{i_{l}}}\right)-\frac{\left(N_{s}\right)}{{\bar{\gamma }}_{i_{k}}}\right)$ and $\beta =-(\sum_{l=1}^{N_{c}-N_{s}}(\frac{1}{{\bar{\gamma }}_{i_{l}}})-\frac{N_{c}-N_{s}}{{\bar{\gamma }}_{i_{h}}})$, we can express the first integral term in Eq (26) as

$$\begin{array}{c} \int_{0}^{\frac{y}{N_{s}}}\int_{\frac{y}{N_{s}}}^{\frac{x}{N_{c}-N_{s}}}\text{exp}\left(\beta z_{2}\right)\text{exp}\left(\alpha z_{4}\right)U\left(x-\left(N_{c}-N_{s}\right)\cdot z_{2}\right)U\left(y-\left(N_{s}\right)\cdot z_{4}\right)dz_{2}dz_{4}\\ =\int_{0}^{\frac{y}{N_{s}}}\int_{\frac{y}{N_{s}}}^{\frac{x}{N_{c}-N_{s}}}\text{exp}\left(\beta z_{2}\right)\text{exp}\left(\alpha z_{4}\right)dz_{2}dz_{4}\\ =I\left(z_{2},\beta ,\frac{y}{N_{s}},\frac{x}{N_{c}-N_{s}};z_{4},\alpha ,0,\frac{y}{N_{s}}\right). \end{array}$$ (28)

Similarly, by replacing *α* with *α*′ in Eq (28) where $\alpha^{\prime }=-\left(\sum_{l=N_{c}-N_{s}+1}^{N_{c}}\left(\frac{1}{{\bar{\gamma }}_{i_{l}}}\right)+\sum_{m=1}^{g}\frac{1}{{\bar{\gamma }}_{i_{{j^{\prime }}_{m}}}}-\frac{\left(N_{s}\right)}{{\bar{\gamma }}_{i_{k}}}\right)$, we can easily obtain the third integral term in Eq (26).

For the second and fourth integral terms, we need a special care on the valid integration region of *z*_2_ and *z*_4_. More specifically, *z*_2_ should satisfy two following conditions

$$\begin{array}{c} z_{2}\leq \frac{x}{N_{c}-N_{s}}\;\text{and}\;z_{2}\leq \frac{y-\left(N_{s}-l\right)z_{4}}{l} \end{array}$$ (29)

which directly lead

$$\begin{array}{c} z_{2}\leq \text{min}\left[\frac{x}{N_{c}-N_{s}},\frac{y-\left(N_{s}-l\right)z_{4}}{l}\right]. \end{array}$$ (30)

Hence, if $\frac{x}{N_{c}-N_{s}}\leq \frac{y-(N_{s}-l)z_{4}}{l}$, we get

$$\begin{array}{c} \frac{y}{N_{s}}<z_{2}<\frac{x}{N_{c}-N_{s}}\;\text{and}\;0<z_{4}<\text{min}\left[\frac{y}{N_{s}},\frac{y-\frac{l}{N_{c}-N_{s}}x}{\left(N_{s}-l\right)}\right]. \end{array}$$ (31)

Otherwise, if $\frac{x}{N_{c}-N_{s}}>\frac{y-(N_{s}-l)z_{4}}{l}$, the valid integral regions for *z*_2_ is

$$\begin{array}{c} \frac{y}{N_{s}}<z_{2}<\frac{y-\left(N_{s}-l\right)z_{4}}{l}, \end{array}$$ (32)

and for *z*_4_, we need to consider both cases

$$\begin{array}{c} 0<z_{4}<\text{min}\left[\frac{y}{N_{s}},\frac{y-\frac{l}{N_{c}-N_{s}}x}{\left(N_{s}-l\right)}\right]\;\text{and}\;0<z_{4}<\frac{y}{N_{s}} \end{array}$$ (33)

by considering the unit step function, $\left\{1-U(\frac{x}{N_{c}-N_{s}}-\frac{y-(N_{s}-l)\cdot z_{4}}{l})\right\}$. As results, we can express the second integral term in Eq (26) as three double-integral terms

$$\begin{array}{c} \int_{0}^{\min\left[\frac{y}{N_{s}},\frac{y-\frac{l}{N_{c}-N_{s}}\cdot x}{\left(N_{s}-l\right)}\right]}\text{exp}\left(-\left(\sum_{l=N_{c}-N_{s}+1}^{N_{c}}\left(\frac{1}{{\bar{\gamma }}_{i_{l}}}\right)-\frac{\left(N_{s}-l\right)}{{\bar{\gamma }}_{i_{k}}}-\sum_{q=1}^{l}\frac{1}{{\bar{\gamma }}_{i_{j_{q}}}}\right)z_{4}\right)\\ \int_{\frac{y}{N_{s}}}^{\frac{x}{N_{c}-N_{s}}}\text{exp}\left(-\left(\sum_{l=1}^{N_{c}-N_{s}}\left(\frac{1}{{\bar{\gamma }}_{i_{l}}}\right)+\sum_{q=1}^{l}\frac{1}{{\bar{\gamma }}_{i_{j_{q}}}}-\frac{N_{c}-N_{s}}{{\bar{\gamma }}_{i_{h}}}-\frac{l}{{\bar{\gamma }}_{i_{k}}}\right)z_{2}\right)dz_{2}dz_{4}\\ +\left\{\int_{0}^{\frac{y}{N_{s}}}\text{exp}\left(-\left(\sum_{l=N_{c}-N_{s}+1}^{N_{c}}\left(\frac{1}{{\bar{\gamma }}_{i_{l}}}\right)-\frac{\left(N_{s}-l\right)}{{\bar{\gamma }}_{i_{k}}}-\sum_{q=1}^{l}\frac{1}{{\bar{\gamma }}_{i_{j_{q}}}}\right)z_{4}\right)\right.\\ \;\;\int_{\frac{y}{N_{s}}}^{\frac{y-\left(N_{s}-l\right)\cdot z_{4}}{l}}\text{exp}\left(-\left(\sum_{l=1}^{N_{c}-N_{s}}\left(\frac{1}{{\bar{\gamma }}_{i_{l}}}\right)+\sum_{q=1}^{l}\frac{1}{{\bar{\gamma }}_{i_{j_{q}}}}-\frac{N_{c}-N_{s}}{{\bar{\gamma }}_{i_{h}}}-\frac{l}{{\bar{\gamma }}_{i_{k}}}\right)z_{2}\right)dz_{2}dz_{4}\\ \;\;-\int_{0}^{\min\left[\frac{y}{N_{s}},\frac{y-\frac{l}{N_{c}-N_{s}}\cdot x}{\left(N_{s}-l\right)}\right]}\text{exp}\left(-\left(\sum_{l=N_{c}-N_{s}+1}^{N_{c}}\left(\frac{1}{{\bar{\gamma }}_{i_{l}}}\right)-\frac{\left(N_{s}-l\right)}{{\bar{\gamma }}_{i_{k}}}-\sum_{q=1}^{l}\frac{1}{{\bar{\gamma }}_{i_{j_{q}}}}\right)z_{4}\right)\\ \;\int_{\frac{y}{N_{s}}}^{\frac{y-\left(N_{s}-l\right)\cdot z_{4}}{l}}\text{exp}\left(-\left(\sum_{l=1}^{N_{c}-N_{s}}\left(\frac{1}{{\bar{\gamma }}_{i_{l}}}\right)+\sum_{q=1}^{l}\frac{1}{{\bar{\gamma }}_{i_{j_{q}}}}-\frac{N_{c}-N_{s}}{{\bar{\gamma }}_{i_{h}}}-\frac{l}{{\bar{\gamma }}_{i_{k}}}\right)z_{2}\right)dz_{2}dz_{4}\}. \end{array}$$ (34)

The fourth integral term in Eq (26) can be also expressed in a quite similar way.

In Eq (34), the closed-form expression of the first double-integral term can be obtained with the help of Eq (27). However, for the second and the third double-integral terms in Eq (34), the inner integral limit depends on the outer variable. Therefore, for these cases, we can obtain the closed-form expression with the help of another useful common function as

$$\begin{array}{c} I^{\prime }\left(x,e,a,b-b^{\prime }y;y,f,c,d\right)\\ =\int_{c}^{d}\text{exp}\left(f\cdot y\right)\int_{a}^{b-b^{\prime }y}\text{exp}\left(e\cdot x\right)dxdy\\ =\frac{1}{e}\left[\text{exp}\left(e\cdot a\right)\frac{1}{\left(f-e\cdot b^{\prime }\right)}\left\{\text{exp}\left(\left(f-e\cdot b^{\prime }\right)\cdot d\right)-\text{exp}\left(\left(f-e\cdot b^{\prime }\right)\cdot c\right)\right\}\right.\\ \;\left.-\text{exp}\left(e\cdot a\right)\frac{1}{f}\left\{\text{exp}\left(f\cdot d\right)-\text{exp}\left(f\cdot c\right)\right\}\right]. \end{array}$$ (35)

Letting $\alpha^{\prime \prime }\;=\;-\left(\sum_{l=N_{c}-N_{s}+1}^{N_{c}}\left(\frac{1}{{\bar{\gamma }}_{i_{l}}}\right)-\frac{\left(N_{s}-l\right)}{{\bar{\gamma }}_{i_{k}}}-\sum_{q=1}^{l}\frac{1}{{\bar{\gamma }}_{i_{j_{q}}}}\right)$ and $\beta^{\prime }\;=\;-(\sum_{l=1}^{N_{c}-N_{s}}(\frac{1}{{\bar{\gamma }}_{i_{l}}})\;+\;\sum_{q=1}^{l}\frac{1}{{\bar{\gamma }}_{i_{j_{q}}}}-\frac{N_{c}-N_{s}}{{\bar{\gamma }}_{i_{h}}}-\frac{l}{{\bar{\gamma }}_{i_{k}}})$, we can finally obtain the closed-form expression of Eq (34) or equivalently the second integral term in Eq (26) as

$$\begin{array}{c} I\left(z_{2},\beta^{\prime },\frac{y}{N_{s}},\frac{x}{N_{c}-N_{s}};z_{4},\alpha^{\prime \prime },0,\min\left[\frac{y}{N_{s}},\frac{y-\frac{l}{N_{c}-N_{s}}\cdot x}{\left(N_{s}-l\right)}\right]\right)\\ \;+I^{\prime }\left(z_{2},\beta^{\prime },\frac{y}{N_{s}},\frac{y-\left(N_{s}-l\right)\cdot z_{4}}{l};z_{4},\alpha^{\prime \prime },0,\frac{y}{N_{s}}\right)\\ \;-I^{\prime }\left(z_{2},\beta^{\prime },\frac{y}{N_{s}},\frac{y-\left(N_{s}-l\right)\cdot z_{4}}{l};z_{4},\alpha^{\prime \prime },0,\min\left[\frac{y}{N_{s}},\frac{y-\frac{l}{N_{c}-N_{s}}\cdot x}{\left(N_{s}-l\right)}\right]\right). \end{array}$$ (36)

For fourth integral term in Eq (26), by replacing *α*″ with *α*‴ in Eq (36) where $\alpha^{\prime \prime \prime }=-(\sum_{l=N_{c}-N_{s}+1}^{N_{c}}\left(\frac{1}{{\bar{\gamma }}_{i_{l}}}\right)+\sum_{m=1}^{g}\frac{1}{{\bar{\gamma }}_{i_{{j^{\prime }}_{m}}}}-\frac{\left(N_{s}-l\right)}{{\bar{\gamma }}_{i_{k}}}-\sum_{q=1}^{l}\frac{1}{{\bar{\gamma }}_{i_{j_{q}}}})$, we can easily obtain the closed-form result.

Then, by substituting all these closed-form results and after some manipulations, we can obtain the final closed-form expression of Eq (16) as

fY,W1x,y=∑iNc,1,⋯,iN1,1iNc,1≠⋯≠iN1,11,2,⋯,N11γ¯iNc,1∑iNc-Ns,1=1iNc-Ns,1≠iNc,1,⋯,iN1,1N11γ¯iNc-Ns,1∑τB,3∈PNs-1IN1-τB,2-τB,4∑k=Nc-Ns+1Nc-1Ck,Nc-Ns+1,Nc-1[∑τB,1∈PNc-Ns-1IN1-τB,2-τB,3-τB,4∑h=1Nc-Ns-1Ch,1,Nc-Ns-1exp-xγ¯ih,1+yγ¯ik,1Iz2,β,yNs,xNc-Ns;z4,α,0,yNs+∑l=1Ns-1-1l∑j1=j0+Nc-Ns+1Nc-l⋯∑jl=jl-1+1Nc-1∑τB,1∈PNc-Ns-1IN1-τB,2-τB,3-τB,4∑h=1Nc-Ns-1Ch,1,Nc-Ns-1exp-xγ¯ih,1+yγ¯ik,1×Iz2,β′,yNs,xNc-Ns;z4,α′′,0,minyNs,y-lNc-Ns·xNs-l+I′z2,β′,yNs,y-Ns-l·z4l;z4,α′′,0,yNs-I′z2,β′,yNs,y-Ns-l·z4l;z4,α′′,0,minyNs,y-lNc-Ns·xNs-l]+∑iNc,1,⋯,iN1,1iNc,1≠⋯≠iN1,11,2,⋯,N11γ¯iNc,1∑g=1N1-Nc-1g∑j′1=j′0+Nc+1N1-g+1⋯∑j′g=j′g-1+1N1∑iNc-Ns,1=1iNc-Ns,1≠iNc,1,⋯,iN1,1N11γ¯iNc-Ns,1∑τB,3∈PNs-1IN1-τB,2-τB,4∑k=Nc-Ns+1Nc-1Ck,Nc-Ns+1,Nc-1[∑τB,1∈PNc-Ns-1IN1-τB,2-τB,3-τB,4∑h=1Nc-Ns-1Ch,1,Nc-Ns-1exp-xγ¯ih,1+yγ¯ik,1×Iz2,β,yNs,xNc-Ns;z4,α′,0,yNs+∑l=1Ns-1-1l∑j1=j0+Nc-Ns+1Nc-l⋯∑jl=jl-1+1Nc-1∑τB,1∈PNc-Ns-1IN1-τB,2-τB,3-τB,4∑h=1Nc-Ns-1Ch,1,Nc-Ns-1exp-xγ¯ih,1+yγ¯ik,1×Iz2,β′,yNs,xNc-Ns;z4,α′′′,0,minyNs,y-lNc-Ns·xNs-l+I′z2,β′,yNs,y-Ns-l·z4l;z4,α′′′,0,yNs-I′z2,β′,yNs,y-Ns-l·z4l;z4,α′′′,0,minyNs,y-lNc-Ns·xNs-l] (37)

where $\alpha =-\left(\sum_{l=N_{c}-N_{s}+1}^{N_{c}}\left(\frac{1}{{\bar{\gamma }}_{i_{l,1}}}\right)-\frac{\left(N_{s}\right)}{{\bar{\gamma }}_{i_{k,1}}}\right)$,

$\alpha^{\prime }=-(\sum_{l=N_{c}-N_{s}+1}^{N_{c}}\left(\frac{1}{{\bar{\gamma }}_{i_{l,1}}}\right)+\sum_{m=1}^{g}\frac{1}{{\bar{\gamma }}_{i_{{j^{\prime }}_{m},1}}}-\frac{\left(N_{s}\right)}{{\bar{\gamma }}_{i_{k,1}}})$,

$\alpha^{\prime \prime }=-\left(\sum_{l=N_{c}-N_{s}+1}^{N_{c}}\left(\frac{1}{{\bar{\gamma }}_{i_{l,1}}}\right)-\frac{\left(N_{s}-l\right)}{{\bar{\gamma }}_{i_{k,1}}-\sum_{q=1}^{l}\frac{1}{{\bar{\gamma }}_{i_{j_{q},1}}}}\right)$,

$\alpha^{\prime \prime \prime }=-(\sum_{l=N_{c}-N_{s}+1}^{N_{c}}\left(\frac{1}{{\bar{\gamma }}_{i_{l,1}}}\right)+\sum_{m=1}^{g}\frac{1}{{\bar{\gamma }}_{i_{{j^{\prime }}_{m},1}}}-\frac{\left(N_{s}-l\right)}{{\bar{\gamma }}_{i_{k,1}}}-\sum_{q=1}^{l}\frac{1}{{\bar{\gamma }}_{i_{j_{q},1}}})$,

$\beta =-(\sum_{l=1}^{N_{c}-N_{s}}(\frac{1}{{\bar{\gamma }}_{i_{l,1}}})-\frac{N_{c}-N_{s}}{{\bar{\gamma }}_{i_{h,1}}})$, and

$\beta^{\prime }=-(\sum_{l=1}^{N_{c}-N_{s}}(\frac{1}{{\bar{\gamma }}_{i_{l,1}}})+\sum_{q=1}^{l}\frac{1}{{\bar{\gamma }}_{i_{j_{q},1}}}-\frac{N_{c}-N_{s}}{{\bar{\gamma }}_{i_{h,1}}}-\frac{l}{{\bar{\gamma }}_{i_{k,1}}})$. Further,

$\tau_{B,1}=\tau_{1}\left(i,1,N_{c}-N_{s}-1\right)=\left\{i_{1,1},i_{2,1},\cdots ,i_{N_{c}-N_{s}-1,1}\right\}$,

$\tau_{B,2}=\tau_{1}\left(i,N_{c}-N_{s},N_{c}-N_{s}\right)=\left\{i_{N_{c}-N_{s},1}\right\}$,

$\tau_{B,3}=\tau_{1}\left(i,N_{c}-N_{s}+1,N_{c}-1\right)=\left\{i_{N_{c}-N_{s}+1,1},i_{N_{c}-N_{s}+2,1},\cdots ,i_{N_{c}-1,1}\right\}$, and

$\tau_{B,4}=\tau_{1}\left(i,N_{c},N_{1}\right)=\left\{i_{N_{c},1},i_{N_{c}+1,1},\cdots ,i_{N_{1},1}\right\}$.

### CDF of the *N*_*c*_/*N*_1_-GSC output SNR over i.n.d. Rayleigh fading, $F_{Y+W_{1}}(x)$

In this case, by replacing *N*_*s*_ and *z*_1_ in [16], Eq (42)] with *N*_*c*_ and *z*−*z*_2_, respectively, and then by applying Eq (25), we can obtain the following double-integral form for Eq (11) as

$$\begin{array}{l} \int_{0}^{x}\;\;\int_{0}^{\frac{z}{N_{c}}}f_{Z}\left(z-z_{2},z_{2}\right)dz_{2}dz=\sum_{i_{N_{c}}=1}^{N}\;\frac{1}{{\bar{\gamma }}_{i_{N_{c}}}}\sum_{\begin{array}{c} i_{N_{c}+1},\cdots ,i_{N}\\ i_{N_{c}+1}\neq \cdots \neq i_{N}\\ \vdots \\ i_{N}\neq i_{N_{c}+1} \end{array}}^{1,2,\cdots N}\sum_{\begin{array}{c} \left\{\;i_{1},\cdots ,i_{N_{c}-1}\;\right\}\in \\ P_{N_{c}\;-\;1}\;\left(\;I_{N}\;-\left\{\;i_{N_{c}}\;\right\}-\left\{\;i_{N_{c}\;+1},\cdots ,i_{N}\;\right\}\;\right) \end{array}}\\ \prod_{\begin{array}{c} q=1\\ \left\{\;i_{1},\cdots ,i_{N_{c}-1}\;\right\} \end{array}}^{N_{c}}\;\;C_{q,1,N_{c}-1}[-\int_{0}^{x}\;\;\int_{0}^{\frac{z}{N_{c}}}\text{exp}\left(-\frac{z}{{\bar{\gamma }}_{i_{q}}}-\left(\sum_{l=1}^{N_{c}}\frac{1}{{\bar{\gamma }}_{i_{l}}}-\frac{N_{c}}{{\bar{\gamma }}_{i_{q}}}\right)z_{2}\right)dz_{2}dz\\ -\prod_{\begin{array}{c} k^{\prime }=1\\ \left\{i_{N_{c}+1},\cdots ,i_{N}\right\} \end{array}}^{N-N_{c}}{\left(-1\right)}^{k^{\prime }}\sum_{j_{1}=j_{0}+N_{c}+1}^{N-k^{\prime }+1}\cdots \sum_{j_{k^{\prime }}=j_{k^{\prime }-1}+1}^{N}\int_{0}^{x}\;\int_{0}^{\frac{z}{N_{c}}}\text{exp}\left(-\frac{z}{{\bar{\gamma }}_{i_{q}}}-\left(\sum_{l=1}^{N_{c}}\frac{1}{{\bar{\gamma }}_{i_{l}}}+\sum_{m=1}^{k^{\prime }}\frac{1}{{\bar{\gamma }}_{i_{j_{m}}}}-\frac{N_{c}}{{\bar{\gamma }}_{i_{q}}}\right)z_{2}\right)dz_{2}dz]. \end{array}$$ (38)

In Eq (38), the first and the second double-integral terms can be expressed in closed form by directly applying Eq (35) as

$$\begin{array}{cc} & \int_{0}^{x}\int_{0}^{\frac{z}{N_{c}}}\text{exp}\left(-\frac{z}{{\bar{\gamma }}_{i_{q}}}\right)\text{exp}\left(-\left(\sum_{l=1}^{N_{c}}\frac{1}{{\bar{\gamma }}_{i_{l}}}-\frac{N_{c}}{{\bar{\gamma }}_{i_{q}}}\right)z{}_{2}\right)dz_{2}dz\\ & =I^{\prime }\left(z_{2},-\left(\sum_{l=1}^{N_{c}}\frac{1}{{\bar{\gamma }}_{i_{l}}}-\frac{N_{c}}{{\bar{\gamma }}_{i_{q}}}\right),0,\frac{z}{N_{c}};z,-\frac{z}{{\bar{\gamma }}_{i_{q}}},0,x\right) \end{array}$$ (39)

and

$$\begin{array}{cc} & \int_{0}^{x}\int_{0}^{\frac{z}{N_{c}}}\text{exp}\left(-\frac{z}{{\bar{\gamma }}_{i_{q}}}\right)\text{exp}\left(-\left(\sum_{l=1}^{N_{c}}\frac{1}{{\bar{\gamma }}_{i_{l}}}+\sum_{m=1}^{k^{\prime }}\frac{1}{{\bar{\gamma }}_{i_{j_{m}}}}-\frac{N_{c}}{{\bar{\gamma }}_{i_{q}}}\right)z{}_{2}\right)dz_{2}dz\\ & =I^{\prime }\left(z_{2},-\left(\sum_{l=1}^{N_{c}}\frac{1}{{\bar{\gamma }}_{i_{l}}}+\sum_{m=1}^{k^{\prime }}\frac{1}{{\bar{\gamma }}_{i_{j_{m}}}}-\frac{N_{c}}{{\bar{\gamma }}_{i_{q}}}\right),0,\frac{z}{N_{c}};z,-\frac{z}{{\bar{\gamma }}_{i_{q}}},0,x\right). \end{array}$$ (40)

Substituting Eqs (39) and (40) in (38), we can obtain the target CDF in closed form as

$$\begin{array}{l} F_{Y+W_{1}}\left(x\right)\;=\;\sum_{i_{N_{c},1}=1}^{N_{1}}\;\frac{1}{{\bar{\gamma }}_{i_{N_{c},1}}}\;\sum_{\begin{array}{c} i_{N_{c}+1,1},\cdots ,i_{N_{1},1}\\ i_{N_{c}+1,1}\neq \cdots \neq i_{N_{1},1}\\ \vdots \\ i_{N_{1},1}\neq i_{N_{c}+1,1} \end{array}}^{1,2,\cdots N_{1}}\sum_{\begin{array}{c} \tau_{A,1}\in \\ P_{N_{c}\;-\;1}\;\left(\;I_{N_{1}}\;-\;\tau_{A,2}\;-\;\tau_{A,3}\;\right) \end{array}}\prod_{\begin{array}{c} q=1\\ \tau_{A,1} \end{array}}^{N_{c}}\;\;C_{q,1,N_{c}-1}\\ \times \;[-I^{\prime }\;\left(\;z_{2},\;-\;\left(\;\sum_{l=1}^{N_{c}}\;\frac{1}{{\bar{\gamma }}_{i_{l}}}-\;\frac{N_{c}}{{\bar{\gamma }}_{i_{q}}}\right),\;0,\;\frac{z}{N_{c}};\;z,\;-\frac{z}{{\bar{\gamma }}_{i_{q}}},\;0,\;x\;\right)\\ -\;\prod_{\begin{array}{c} k^{\prime }=1\\ \tau_{A,3} \end{array}}^{N_{1}-N_{c}}\;\;{\left(-1\right)}^{k^{\prime }}\;\sum_{j_{1}=j_{0}+N_{c}+1}^{N_{1}-k^{\prime }+1}\;\cdots \;\sum_{j_{k^{\prime }}=j_{k^{\prime }-1}+1}^{N_{1}}\;I^{\prime }\;\left(\;z_{2},\;-\;\left(\;\sum_{l=1}^{N_{c}}\;\frac{1}{{\bar{\gamma }}_{i_{l}}}+\;\sum_{m=1}^{k^{\prime }}\frac{1}{{\bar{\gamma }}_{i_{j_{m}}}}-\;\frac{N_{c}}{{\bar{\gamma }}_{i_{q}}}\right)\;,\;0,\;\frac{z}{N_{c}};z,-\frac{z}{{\bar{\gamma }}_{i_{q}}},\;0,\;x\;\right)], \end{array}$$ (41)

where *j*_0_ = 0 and

$$\begin{array}{c} C_{l,n_{1},n_{2}}=\frac{1}{\prod_{l=n_{1}}^{n_{2}}\left(-{\bar{\gamma }}_{i_{l,1}}\right)F_{l,n_{1},n_{2}}^{\prime }\left(\frac{1}{{\bar{\gamma }}_{i_{l,1}}}\right)} \end{array}$$ (42)

where

$$\begin{array}{ll} F_{l,n_{1},n_{2}}^{\prime }\left(x\right)\;= & \left[\overset{n_{2}-n_{1}}{\underset{l=1}{\sum }}\;\left(n_{2}\;-\;n_{1}\;-\;l\;+\;1\right)x^{n_{2}-n_{1}-l}{\left(-1\right)}^{l}\overset{n_{2}-l+1}{\underset{j_{1}=j_{0}+n_{1}}{\sum }}\;\cdots \;\overset{n_{2}}{\underset{j_{l}=j_{l-1}+1}{\sum }}\overset{l}{\underset{m=1}{\prod }}\frac{1}{{\bar{\gamma }}_{i_{j_{m},1}}}\;\right]\\ & +\;\left(n_{2}-n_{1}+1\right)x^{n_{2}-n_{1}}. \end{array}$$ (43)

Further, $\tau_{A,1}=\tau_{1}\left(i,1,N_{c}-1\right)=\left\{i_{1,1},i_{2,1},\cdots ,i_{N_{c}-1,1}\right\}$, $\tau_{A,2}=\tau_{1}\left(i,N_{c},N_{c}\right)=\left\{i_{N_{c},1}\right\}$, and $\tau_{A,3}=\tau_{1}\left(i,N_{c}+1,N_{1}\right)=\left\{i_{N_{c}+1,1},i_{N_{c}+2,1},\cdots ,i_{N_{1},1}\right\}$.

### CDF of the sums of *N*_*s*_ strongest paths from each target BS over i.n.d. Rayleigh fading, $F_{W_{n}}(x)$

In this case, we can easily obtain the closed-form expression of Eq (17) by directly adopting the derived result in Eq (11), specifically replacing *N*_*c*_ with *N*_*s*_, as

$$\begin{array}{l} F_{W_{n}}\left(x\right)=\;\sum_{i_{N_{s},n}=1}^{N_{n}}\;\frac{1}{{\bar{\gamma }}_{i_{N_{s},n}}}\;\sum_{\begin{array}{c} i_{N_{s}+1,n},\cdots ,i_{N_{n},n}\\ i_{N_{s}+1,n}\neq \cdots \neq i_{N_{n},n}\\ \vdots \\ i_{N_{n},n}\neq i_{N_{s}+1,n} \end{array}}^{1,2,\cdots N_{n}}\sum_{\begin{array}{c} \tau_{C,1}\in \\ P_{N_{s}\;-\;1}\;\left(\;I_{N_{n}}\;-\;\tau_{C,2}\;-\;\tau_{C,3}\;\right) \end{array}}\prod_{\begin{array}{c} q=1\\ \tau_{C,1} \end{array}}^{N_{s}}C_{q,1,N_{s}-1}\;\\ \times \;[-\;I^{\prime }\;\left(\;z_{2},-\;\left(\;\sum_{l=1}^{N_{s}}\;\frac{1}{{\bar{\gamma }}_{i_{l}}}-\;\frac{N_{s}}{{\bar{\gamma }}_{i_{q}}}\;\right),\;0,\;\frac{z}{N_{s}};\;z,-\frac{z}{{\bar{\gamma }}_{i_{q}}},\;0,\;x\;\right)\;\\ -\;\prod_{\begin{array}{c} k^{\prime }=1\\ \tau_{C,3} \end{array}}^{N_{n}-N_{s}}\;\;{\left(-1\right)}^{k^{\prime }}\;\sum_{j_{1}\;=\;j_{0}\;+\;N_{s}\;+\;1}^{N_{n}\;-\;k^{\prime }\;+\;1}\;\cdots \;\sum_{j_{k^{\prime }}=j_{k^{\prime }-1}+1}^{N_{n}}\;\;I^{\prime }\;\left(\;z_{2},-\;\left(\;\sum_{l=1}^{N_{s}}\;\frac{1}{{\bar{\gamma }}_{i_{l}}}\;+\;\sum_{m=1}^{k^{\prime }}\;\frac{1}{{\bar{\gamma }}_{i_{j_{m}}}}-\;\frac{N_{s}}{{\bar{\gamma }}_{i_{q}}}\;\right)\;,0,\;\frac{z}{N_{s}};\;z,-\frac{z}{{\bar{\gamma }}_{i_{q}}},\;0,\;x\;\right)\;], \end{array}$$ (44)

where $\tau_{C,1}=\tau_{n}\left(i,1,N_{s}-1\right)=\left\{i_{1,n},i_{2,n},\cdots ,i_{N_{s}-1,n}\right\}$, $\tau_{C,2}=\tau_{n}\left(i,N_{s},N_{s}\right)=\left\{i_{N_{s},n}\right\}$, and $\tau_{C,3}=\tau_{n}\left(i,N_{s}+1,N_{1}\right)=\left\{i_{N_{s}+1,n},i_{N_{s}+2,n},\cdots ,i_{N_{1},n}\right\}$.

Note that while [2] provides the non-closed-form expressions even over i.i.d. fading assumptions since the final results involve finite integrations, in this report we provide all three required key statistics in Eqs (37), (41) and (44) in closed form to accurately investigating the performance measures. With these derived joint statistics, the outage probability as well as other performance measures mentioned in the ‘System Models and Performance Measures’ section, can be easily calculated with standard mathematical softwares such as Mathematica.

## Simulation results and conclusions

In this work, we studied the assessment tool for the finger replacement scheme proposed in [2] over i.n.d. fading conditions by providing the general comprehensive mathematical framework. Specifically, we provided the closed-form expressions for the required key statistics of i.n.d. ordered exponential RVs by applying the unified framework proposed in [15, 16]. Also, the general comprehensive frameworks for the outage performance based on these statistical results are shown.

**Fig 2:**
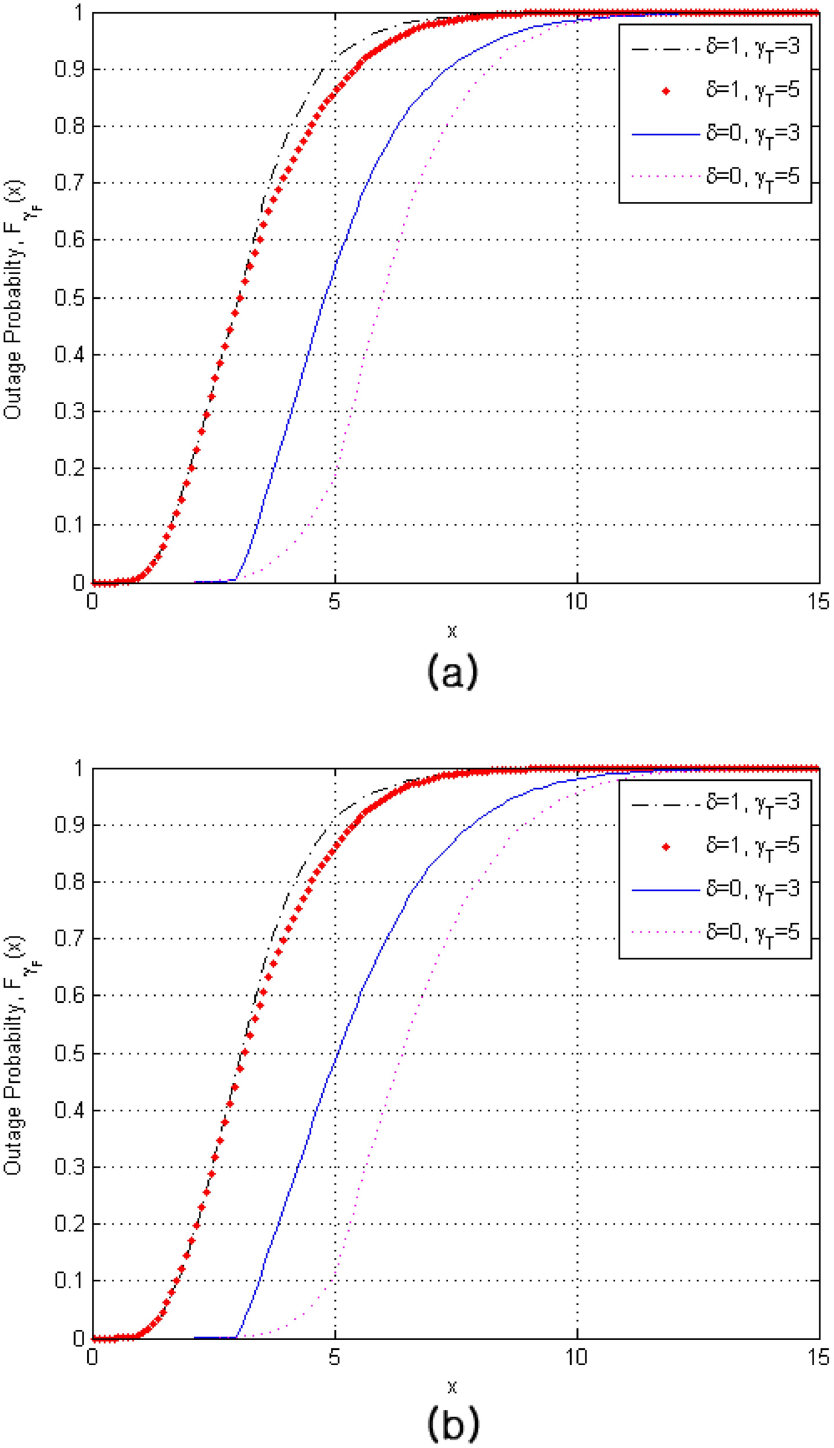
Outage probability of finger replacement schemes for RAKE receivers over i.n.d. Rayleigh fading channels when *L* = 4, *N*_1_ = ⋯ = *N*_4_ = 5, and *N*_*c*_ = 3. (a) *N*_*s*_ = 1 (b) *N*_*s*_ = 2.

In Fig 2, we assess the effect of non-identically distributed paths on the outage performance of the replacement schemes. The comparative analysis results are shown in Table 1. More specifically, instead of the uniform PDP, we now consider an exponentially decaying PDP. Experimental measurements indicate that the radio channel is characterized by an exponentially decaying multipath intensity profile (MIP) for indoor office buildings [19] as well as urban [20] and suburban areas [21]. We assume that the channel has an exponential MIP, for which ${\bar{\gamma }}_{i}=\bar{\gamma }\cdot \text{exp}(-\delta (i-1))$, (1 ≤ *i* ≤ *N*_*n*_, 1 ≤ *n* ≤ *L*) where ${\bar{\gamma }}_{i}$ is the average SNR of the *i*-th path out of the total available resolvable paths from each BS, $\bar{\gamma }$ is the strongest average SNR (or the average SNR of the first path), and *δ* is the average fading power decay factor where *δ* = 0 means identically distributed paths. These results show that the effect of path unbalance induces non-negligible performance degradation compared with the results for i.i.d. fading scenario. This effect must be taken into account for the accurate prediction of the performance over i.n.d. fading environments and, with our analytical results, we believe that our results make it available more easily. In addition, we can also observe from this figure that the bigger the size of the comparison block, *N*_*s*_, the better the performance, especially when *δ* = 0 while the difference of the outage performance is negligible when *δ* = 1. This is because there is a higher chance to have a better group among all *W*_*n*_ over an i.i.d. assumption but in case of i.n.d. environment, the difference in channel quality among all *W*_*n*_ is relatively small. Note that *W*_1_ is the sum of the *N*_*s*_ smallest paths among the *N*_*c*_ currently used paths from the serving BS while *W*_*n*_(2 ≤ *n* ≤ *L*) are the sums of the *N*_*s*_ strongest paths from each target BS.

**Table 1:** Outage probability (*F*_*γ*_*F*__(*x*)) when *L* = 4, *N*_1_ = ⋯ = *N*_4_ = 5, and *N*_*c*_ = 3.

x	(a) *N*_*s*_ = 1				(b) *N*_*s*_ = 2			
	*δ* = 1 *γ*_*T*_ = 3	*δ* = 1 *γ*_*T*_ = 5	*δ* = 0 *γ*_*T*_ = 3	*δ* = 0 *γ*_*T*_ = 5	*δ* = 1 *γ*_*T*_ = 3	*δ* = 1 *γ*_*T*_ = 5	*δ* = 0 *γ*_*T*_ = 3	*δ* = 0 *γ*_*T*_ = 5
0	0.000	0.000	0.000	0.000	0.000	0.000	0.000	0.000
1	0.014	0.014	0.000	0.000	0.008	0.009	0.000	0.000
2	0.211	0.201	0.000	0.000	0.171	0.170	0.000	0.000
3	0.508	0.499	0.018	0.009	0.491	0.471	0.013	0.003
4	0.790	0.725	0.282	0.061	0.778	0.718	0.253	0.025
5	0.920	0.863	0.562	0.201	0.914	0.864	0.495	0.122
6	0.970	0.944	0.762	0.518	0.964	0.946	0.697	0.411
7	0.988	0.979	0.878	0.760	0.987	0.982	0.835	0.651
8	0.995	0.992	0.938	0.888	0.996	0.993	0.915	0.811
9	0.998	0.997	0.972	0.954	0.998	0.997	0.959	0.907
10	1.000	0.999	0.986	0.982	0.999	0.999	0.980	0.959
11	1.000	1.000	0.993	0.994	1.000	1.000	0.991	0.982
12	1.000	1.000	0.997	0.997	1.000	1.000	0.996	0.993
13	1.000	1.000	0.999	0.999	1.000	1.000	0.998	0.997
14	1.000	1.000	1.000	1.000	1.000	1.000	0.999	0.999
15	1.000	1.000	1.000	1.000	1.000	1.000	1.000	1.000

**Fig 3:**
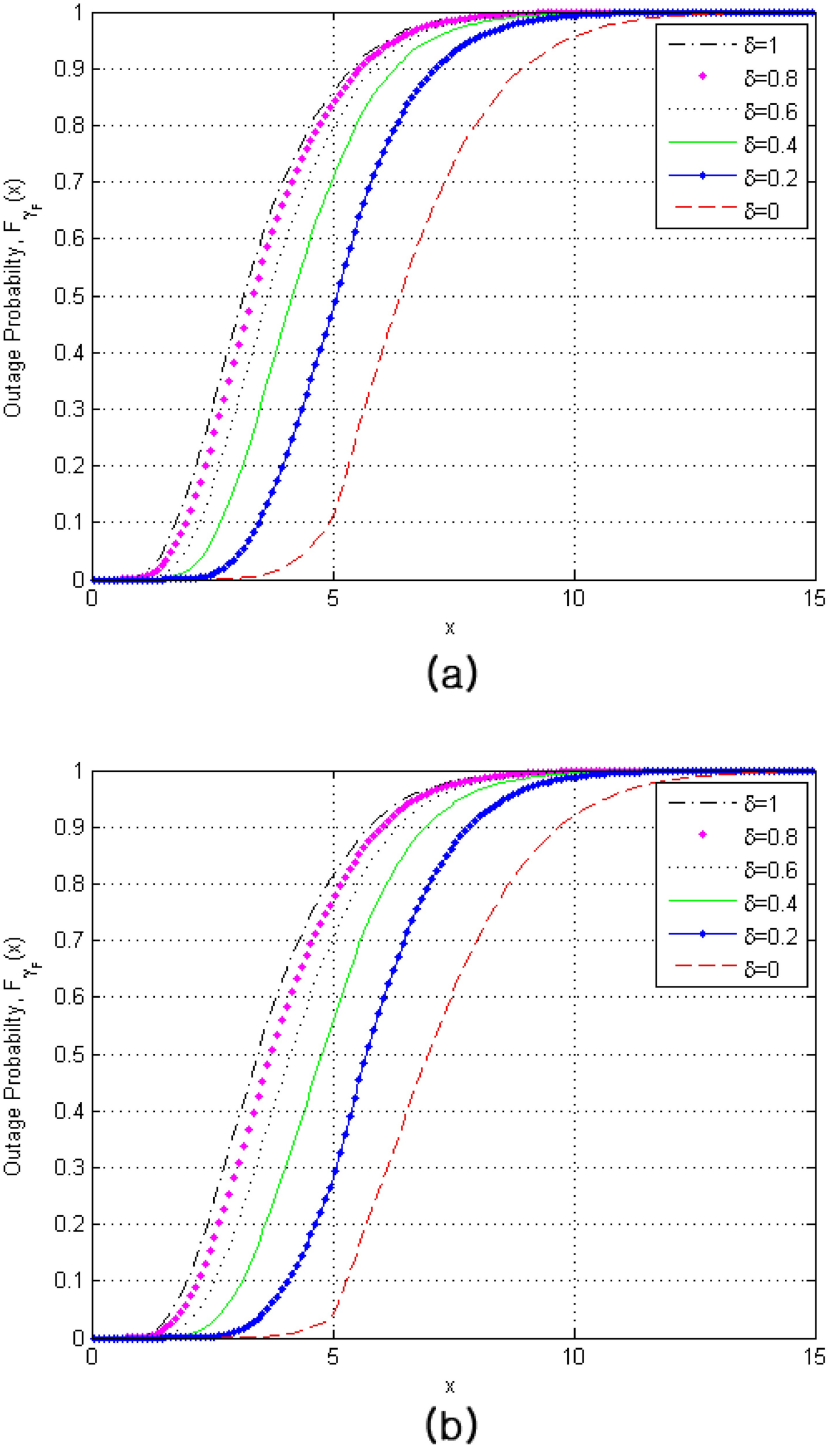
Outage probability of finger replacement schemes for RAKE receivers in the soft handover region for various values of *δ* when *L* = 4, *N*_1_ = ⋯ = *N*_4_ = 5, *N*_*s*_ = 2, and *γ*_*T*_ = 5. (a) *N*_*c*_ = 3 (a) *N*_*c*_ = 4.

Fig 3 shows the outage probability for various values of *δ* and *N*_*c*_. The comparative analysis results are shown in Table 2. As expected, the increase of the number of combined paths, *N*_*c*_, will provide the better performance. However, as *δ* increases, the performance difference is reduced because of the same reason mentioned in Fig 2 such that the increment of the combined signal strength can be relatively smaller compared with that for small *δ*.

**Table 2:** Outage probability (*F*_*γ*_*F*__(*x*)) for various values of *δ* when *L* = 4, *N*_1_ = ⋯ = *N*_4_ = 5, *N*_*s*_ = 2, and *γ*_*T*_ = 5.

x	(a) *N*_*c*_ = 3						(b) *N*_*c*_ = 4					
	*δ* = 1	*δ* = 0.8	*δ* = 0.6	*δ* = 0.4	*δ* = 0.2	*δ* = 0	*δ* = 1	*δ* = 0.8	*δ* = 0.6	*δ* = 0.4	*δ* = 0.2	*δ* = 0
0	0.000	0.000	0.000	0.000	0.000	0.000	0.000	0.000	0.000	0.000	0.000	0.000
1	0.009	0.003	0.001	0.000	0.000	0.000	0.004	0.001	0.000	0.000	0.000	0.000
2	0.170	0.123	0.067	0.020	0.001	0.000	0.116	0.074	0.032	0.006	0.000	0.000
3	0.471	0.413	0.320	0.183	0.044	0.003	0.389	0.309	0.209	0.090	0.013	0.000
4	0.718	0.681	0.607	0.468	0.221	0.025	0.652	0.584	0.480	0.309	0.099	0.006
5	0.864	0.843	0.801	0.718	0.489	0.122	0.821	0.779	0.712	0.568	0.293	0.048
6	0.946	0.936	0.918	0.877	0.754	0.411	0.924	0.903	0.870	0.791	0.597	0.282
7	0.982	0.977	0.969	0.952	0.892	0.651	0.971	0.963	0.947	0.911	0.807	0.516
8	0.993	0.991	0.988	0.981	0.957	0.811	0.988	0.985	0.979	0.966	0.919	0.713
9	0.997	0.997	0.996	0.993	0.984	0.907	0.995	0.994	0.992	0.987	0.969	0.844
10	0.999	0.999	0.998	0.997	0.994	0.959	0.998	0.998	0.998	0.995	0.988	0.923
11	1.000	1.000	0.999	0.999	0.998	0.982	0.999	0.999	0.999	0.998	0.996	0.964
12	1.000	1.000	1.000	1.000	0.999	0.993	1.000	1.000	1.000	0.999	0.999	0.983
13	1.000	1.000	1.000	1.000	1.000	0.997	1.000	1.000	1.000	1.000	0.999	0.992
14	1.000	1.000	1.000	1.000	1.000	0.999	1.000	1.000	1.000	1.000	1.000	0.997
15	1.000	1.000	1.000	1.000	1.000	1.000	1.000	1.000	1.000	1.000	1.000	1.000

In summary, we clearly see from these results the importance of the non-identical fading channel effect on the performance. Based on above results, our analytical results can help the system designer to predict the performance and to design the system (e.g., the number of combined paths and so on) accordingly in order to reduce the effect of decay exponents before applying it to the practical environment.

Our results are much simpler than the multiple-fold integral form based on the conventional MGF approach. Moreover, while it is almost impossible to estimate the required performance measure accurately especially for a large number of resolvable paths even with the conventional mathematical tools, our derived closed-form results, even if they look somewhat messy, make the probabilistic analysis available numerically with the conventional mathematical tools. Note that the slightly modified mathematical analytical framework suitable for the derived joint statistical results can be configured to be directly applicable to other various fading scenarios while the conditional PDF based approach and related results in the previous work was limited only to i.i.d. Rayleigh fading assumptions. Finally, as investigated in [22, 23], with the approximation technique such as a central limit theorem, our results can be further generalized to the case of dependent RVs. However, since a thorough discussion on this is not suitable for this work and beyond the scope of this paper, we will consider this approximation in an analytical sense as a future work.
